# ‘Getting the vaccine makes me a champion of it’: Exploring perceptions towards peer‐to‐peer communication about the COVID‐19 vaccines amongst Australian adults

**DOI:** 10.1111/hex.13751

**Published:** 2023-05-03

**Authors:** Joshua Karras, Mia Harrison, Holly Seale

**Affiliations:** ^1^ School of Population Health University of New South Wales Sydney New South Wales Australia; ^2^ Centre for Social Research in Health (CSRH) University of New South Wales Kensington New South Wales Australia

**Keywords:** attitudes, communication, COVID‐19, immunisation, pandemic, vaccine

## Abstract

**Objectives:**

Peer‐to‐peer communication approaches have been previously described as the ‘power of personal referral’. Rather than relying on official channels of information, peer‐to‐peer communication may have a role in supporting changes in understanding and possibly behaviours. However, in emergency or pandemic situations, there is currently limited understanding of whether community members feel comfortable speaking about their vaccine experiences or advocating to others. This study explored the perceptions of COVID‐19 vaccinated and unvaccinated Australian adults regarding their preferences and opinions about peer‐peer communication and other vaccine communication strategies.

**Study Design:**

Qualitative interview research.

**Methods:**

In‐depth interviews were conducted in September 2021 with 41 members of the Australian community. Thirty‐three participants self‐identified as being vaccinated against COVID‐19, while the remainder were not vaccinated at the time or did not intend on receiving a COVID vaccine.

**Results:**

Amongst those who were vaccinated, participants spoke about being willing to promote the vaccine and correct misinformation and felt empowered following their vaccination. They highlighted the importance of peer‐to‐peer communication and community messaging, expressing the need for both strategies in an immunisation promotional campaign, with a slight emphasis on the persuasive power of communication between family and friends. However, those who were unvaccinated tended to dismiss the role of community messaging, commenting on a desire not to be like one of the many who listened to the advice of others.

**Conclusion:**

During emergency situations, governments and other relevant community organisations should consider harnessing peer‐to‐peer communication amongst motivated individuals as a health communication intervention. However further work is needed to understand the support that this constituent‐involving strategy requires.

**Patient or Public Contribution:**

Participants were invited to participate through a series of online promotional pathways including emails and social media posts. Those who completed the expression of interest and met the study criteria were contacted and sent the full study participant information documentation. A time for a 30 min semi‐structured interview was set and provided with a $50 gift voucher at the conclusion.

## 1 INTRODUCTION

During the COVID‐19 pandemic, communities have witnessed an influx in educational and promotional campaigns for the approved COVID‐19 vaccines. Various communication mechanisms have been implemented to reach community members, including some approaches not traditionally relied upon to promote immunisation in high‐resource settings. One such approach is peer‐to‐peer communication (commonly referred to as word of mouth), which is defined as the ‘power of the personal referral on behaviour’.^1^ Peer‐to‐peer communication explains how individuals of equal standing engage in discourse around a topic. Peer‐to‐peer communication has been previously documented to influence health outcomes, providing an opportunity to peer model^2^ and has been utilised in various contexts across the health industry, particularly within mental health.^3^, ^4^ For this study, we define ‘peer‐to‐peer communication’ as communication between community members, as opposed to communication between health professional members or community leaders and community members. As peer‐to‐peer communication includes a standard dialogue discussion, it can and does occur in all settings where two or more people are together and able to speak in person, over the phone, virtually or otherwise. This may include people communicating unregulated shared and private opinions as well as information based on fact and feeling. In the current context, it is suggested that although the public mass media‐based conversations about COVID‐19 and the associated vaccine have been slowly declining, other forms of communication continue to occur in private and social settings (parties, public transport, around the dinner table etc.).^4^ Conversations about COVID vaccination can occur in a range of relationships, including family, friends, colleagues, neighbours, members of a religious, sporting, social or volunteering community and strangers alike. The range of conversations and the potential impact on behaviour has been previously explored in other public health settings. For example, one study analysed the impact of peer‐to‐peer communication on weight management and the exploration of dietary information in online community forums.^5^ The authors suggested that active participation in peer‐to‐peer communication both discouraged unhealthy eating habits and promoted the individual's self‐efficacy to practice healthy eating habits.

Research has suggested that while mass communication strategies may positively affect vaccination uptake when implemented as a singular intervention, poor, mis, mal and/or dis communication can undermine acceptance within the administered population.^6^ This is especially true within social media, which acted as a primary source of information during the COVID‐19 pandemic.^6^ It is suggested that the enhancement of community perceptions towards vaccines through effective communication interventions as part of a combination approach of peer and medical expert engagement with a vaccine‐hesitant individual, renders the greatest likelihood of vaccine uptake.^7^ For example, a study focused on human papillomavirus vaccination of women in college found that the rate of immunisation almost doubled in the test group, who received a peer‐expert narrative intervention, compared to the control group.^7^ Similarly, a study that presented 312 cancer patients and survivors with a mix of website, print and interpersonal interventions regarding updates on cancer research found that both information retention and motivation to share health information increased compared to the control group.^8^

In the context of COVID‐19 and the need to promote pandemic‐specific public health recommendations, it has been postulated that there is a need to utilise peer‐to‐peer communication within the context of fostering mass perception from a bottom‐up, community lead approach.^9^ However, it is critical that there is a solid understanding of how to engage with community‐level communication strategies effectively, as these strategies have the potential to be associated with both accurate information but also misinformation, disinformation and unsubstantiated anecdotal stories about vaccines.^4^, ^10^ The potential for word‐of‐mouth or peer‐to‐peer communication as a means of disseminating negative information was recently explored in a paper seeking to identify the factors which influence the impact of this style of communication on recipients. The authors found that the primary factors of influence include the message content, the source characteristics and the receiver characteristics.^11^ Peer‐to‐peer communication presents a channel for disseminating information about vaccines, potentially supporting messages targeting hard‐to‐reach groups, including highly vaccine‐hesitant groups who may be less responsive to mass media or community messaging. However, the willingness of community members to talk to their peers about vaccination and the potential role this form of communication could have in supporting vaccine‐hesitant individuals to receive a vaccine during a pandemic or disease outbreak is understudied. Within the Australian context, research to assess the dissemination of vaccine information on Facebook from 1 December 2020 to 28 February 2022 determined that both the censoring of vaccine misinformation as an attempt to improve immunisation rates did not only reduce vaccine‐centric narratives but antagonised users and induced migration to less monitored platforms which contain more radical perceptions of vaccines.^12^ Another study sought to understand the communication needs of Australian adults regarding vaccines and determined a number of key recommendations, including the importance of addressing concerns about side effects, sharing true, personalised messages and the need to ensure trust and transparency.^13^ There is currently a lack of understanding regarding if and how such recommendations can apply in the peer‐to‐peer setting as opposed to the top‐down or provider‐to‐individual dynamic. To address this gap, we conducted semi‐structured interviews with vaccinated and unvaccinated Australian adults to discuss their preferences and opinions about peer‐peer communication and other vaccine communication strategies and engagement, based on community preferences, with a primary focus to determine if a vaccinated individual experienced any changes in motivations to discuss vaccines as a result of becoming vaccinated.

## 2 METHODS

### 2.1 Study design

Semi‐structured in‐depth telephone interview research was undertaken with COVID‐19 vaccinated and unvaccinated individuals.

### 2.2 Setting and context

The interviews were conducted entirely virtually (either via video conferencing or telephone). This approach supported the inclusion of individuals living outside of Sydney and minimised the risk of exposure to COVID‐19, especially for those who were unvaccinated. Participants were selected based on ensuring adequate distribution of gender and age (18–80 years). There was also representation from every state and territory in Australia, with a few participants maintaining Australian residency or citizenship but taking their interviews overseas. At the time of the interviews, the national adult COVID‐19 vaccination rate was approximately 45%–50%.^14^ As many parts of Australia were experiencing lockdown, the national and political discourse at the time included the potential for vaccinated individuals to enjoy a relaxation of the public health orders.^15^ State health department officials reported local, national and international statistics relating to the pandemic in daily briefings.

### 2.3 Participants and sampling

This qualitative study recruited participants opportunistically via an expression of interest survey posted via different social media channels. Eligible participants included Australian adults (currently residing in‐country or overseas) or temporary visa holders living in Australia over 18. We had participants who did not intend to receive any COVID‐19 vaccine and those who were already vaccinated. Participants were purposefully selected based on age, gender, location, vaccine status and the ability to provide consent. A higher ratio of vaccinated participants was chosen because the primary focus of the study was to investigate perceptions towards peer‐to‐peer communication.

### 2.4 Data collection

A trained data collector conducted the interviews using a pretested semi‐structured interview guide. The interviews were conducted from 4 September 2021 to 6 October 2021, and interview times varied from 15 to 45 min, depending on the organic flow of the conversation as well as the conciseness of the participant. The semi‐structured interviews covered sentiments towards vaccination desires to communicate vaccination, effects of peer‐to‐peer communication and perspectives on best practice communication interventions. A separate guide was used for unvaccinated participants, which included questions intended to understand the potential receptiveness that unvaccinated individuals may have when listening to vaccinated people advocate the COVID‐19 vaccine. Interviews were audio‐recorded with participant consent, and participants were offered a $40 gift card after the interview as reimbursement for their time and contribution. It is suggested that by the aim of the study, the sample specificity, the quality of the retrieved dialogue and the analysis, the information power is sufficiently high to derive the outlined findings in the study results and discussion section.^16^, ^17^

### 2.5 Data analysis

An inductive approach to analysis was utilised with relevant thematic concerns determined and coded with NVIVO12. Such an approach allowed for the ability to synthesise the raw data and establish links with the research objectives and the potential for unexpected findings.^18^ Codes and concepts were derived through open coding of interview transcripts, which were then tested and consolidated into higher‐order themes. Initial coding was conducted by Author 1 and independently reviewed by Authors 2 and 3, with codes, concepts and higher‐order themes discussed and approved by all authors. The first author is a PhD Candidate with expertise and experience in public health policy and global health strategy in communication. Authors 2 and 3 have in‐depth expertise in qualitative research methodologies about public health research, including immunisation intervention strategies.

## 3 RESULTS

One thousand two hundred and eighty‐one people returned an expression of interest to participate, of which forty interviews were undertaken. The characteristics of the interviewees are described in Table 1 using the CORE‐Q reporting format (17).

**Table 1:** Basic information of participants.

Characteristics	% (*N*)
Gender	
Male	55 (22)
Female	45 (18)
Age range	
18–25	15 (6)
26–32	27.5 (11)
33–40	20 (8)
41–48	7.5 (3)
49–56	12.5 (5)
57–64	5 (2)
65–72	7.5 (3)
73–80	5 (2)
81 And above	0
Vaccination status	
Vaccinated	80 (32)
Unvaccinated	20 (8)

### 3.1 Community and government messages go hand in hand

Participants were asked questions that sought to understand the effect of communicating with people from their family or close social networks about COVID‐19 vaccination and how their levels of influence compared to receiving messages from the government, health officials or community leaders. Responses were diverse, with participants citing various considerations when contemplating a response reply, including how close they were with members of their network, what they had heard about the vaccine from others and how fearful they were of contracting the virus.

When participants reflected on the influence that close friends and family members may have, they acknowledged that both peers and health department/officials play a role in communicating and that it ‘really depends on the person’.

I think they [community messaging and word of mouth] both go hand in hand. I think that different people react and respond differently to different authorities. (Bruce, aged 26–32, vaccinated)

This concept aligned closely with the broader perception that the strength of the effect that family and friends could have over an individual's decision‐making process to become vaccinated would rely on the strength and closeness of the relationship.

I think your biggest influences in life would be the people close to you, right? I think it would be stupid to be like, ‘Oh yeah, you know if the government told me too, but my closest my parents and my siblings and all my friends were saying no’. (Starla, aged 57–65, vaccinated)

Unvaccinated participants tended to frame their responses around ‘myself vs. others’, suggesting that while both promotional pathways may work on some, it is less effective for themselves. They tended to dismiss the role of community messaging, commenting on a desire not to be like one of the many who listened to the advice of others. There were several mentions of arguments and disagreements that had occurred between participants and family and friends who disagreed with their decision not to vaccinate.

Whatever they hear the 11:00 briefings, and they'll take it as Bible. They have a big impact on a big chunk of the population; I'd; I'd say probably 60% but me. But for me, No, I listen to it, then I speak to my doctors, and I've got friends who are pharmacists and I and I get my advice. (Harrison, aged 49–56, unvaccinated)

I had a big argument with my sister over it. I was adamant […] I did not want any of my sisters or my parents getting it. (Elizabeth, aged 41–48, unvaccinated)

These arguments appeared to represent a difficult time or relationship breakdown, which elicited an emotional response. Some described a sense of confusion and anger regarding the vaccine resulting in a detrimental effect on their professional and personal lives.

### 3.2 Becoming an advocate of the vaccine

Many participants reported a definitive change in their desire to promote the COVID‐19 vaccine because of receiving it. The enthusiastic sentiment of their responses was explained mainly due to an understanding that they felt more empowered to endorse the vaccine through personal experience and an ability to ‘talk the talk’ since they have ‘walked the walk’. Individuals also made mention of their newfound capacity to ‘practice what is preached’ and that they ‘absolutely felt more empowered’. Many made explicit mention that their change in motivation had already translated into action through an increased number of conversations about vaccines because they had an experience to share.

I felt more authority to talk about it. There was no point in telling people to get vaccinated if I hadn't got vaccinated. (Nicole, aged 26–32, vaccinated)

This was also met with a sense of excitement and relief that vaccinated participants were now significantly less likely to become seriously ill if they were to contract the disease. Other participants reported no change in their desire to communicate the vaccine after receiving it. They expressed a want to complete their duty as a local citizen and just ‘move on’.

No, I'm trying not to let it be anything more than I've done the right thing. I've followed the health orders, and I've gotten vaccinated to protect myself and my family from the virus, and I don't need to plaster it on social media. (Sierra, aged 26–32, vaccinated)

When asked if there had been any changes in their desires or motivation to correct any misinformation about the COVID‐19 vaccine since becoming vaccinated, most vaccinated participants reported a positive change, relaying, in particular, their increased desire to dispel information relating to potential side effects.

Many described sentiments of hesitation in encountering alleged fragments of misinformation, noting the difficulty in advocating for a subject they are ‘not an expert in’. Participants expressed their intention not to get involved in conversations that resulted in the unvaccinated parties of the conversation becoming aggressive or offended in their thoughts on the topic. Some also mentioned a preference not to hear about the misinformed opinions of some of their personal friends who were against vaccination. Others expressed a desire to explain what had occurred to them as a case study and depicted a desire to share their own experience to encourage others to become vaccinated.

It's sort of like, you have to lead by example, so it's very hard to tell someone what their science says before you've actually done it yourself. And once you've done it yourself, you can practice what you preach in some respects. So I think that's made me more confident in that sense, correcting misinformation and disinformation and so forth. (Cole, aged 25–32, vaccinated)

I do feel that I would be more passionate. About saying that you know describing that is OK, provided you know, since I've had a relatively positive reaction to it and haven't had any serious side effects, making me more confident that I could. (Bruce, aged 18–25, vaccinated)

I think going through the process and seeing how easy it was, uhm; it does make me want to get everybody else on board. (Kira, aged 26–32, vaccinated)

However, some participants expressed the opinion that because they felt they had simply completed their citizenry duty of becoming immunised, there was no desire for them to communicate or engage with the topic.

No. It hasn't, it's just I've done. I've done what I needed to do, and hopefully I don't get it, and that's it, you know, now it's up to the vaccine to do it, you know. (Ben, aged 65–72, vaccinated)

When asked to describe the circumstances under which they would speak to others about the COVID‐19 vaccine, individuals expressed varying sentiments of being happy to communicate with anyone, with those who asked, or with groups of people at a certain level of closeness to them and so forth. There was a general tone of openness and willingness to discuss their circumstances and offer advice that aligned with what they had heard or learned firsthand. Some participants noted that they would remain reluctant to do anything more than personal experience and facts only.

Yeah, I guess the only thing that I can say is just my personal experience. That's the only thing that I could add to the boat. (Starla, aged 73–80, vaccinated)

Umm I would like, I would tell them the effectiveness of this vaccine is quite high, especially in preventing severe symptoms and death. (Su Hwan, aged 49–56, vaccinated)

I would ask them like you know what? What are their concerns? What are, why do they feel hesitant or reluctant to get that vaccine? (Fatima, aged 26–32, vaccinated)

The desire to avoid confrontation when discussing the topic of immunisation was present in many participants, including a wish not to ‘force the topic of vaccination’ or make any individuals feel belittled, dismissed or ignored. There was a pattern of wishing to defer to a primary healthcare provider when questions could not be answered or if the conversation was becoming overly emotional.

I generally would want to avoid confrontation and wouldn't want to be the one trying to convince someone to do it. And if I needed to, I probably just encourage them to talk to their GP. (Bruce, aged 18–25, vaccinated)

A few participants expanded on the criteria or theoretical conditions which needed to be met in a situation before discussing vaccines. These included a safe space, knowing the person or feeling that the case permitted an open discussion without fear of backlash.

If someone else is talking about the vaccination, even if they don't ask me specifically. If I think the moment is appropriate, I might say, ‘Oh yeah, no. This is what I got. This is my experience’. (Terrence, aged 26–32, vaccinated)

Some interviewees provided their thoughts on the importance of maintaining a sense of trust, respect, and openness to discussing the decision to vaccinate. They also mentioned the potentially detrimental side effects of mishandling an interaction with a vaccine‐hesitant individual.

Uh like‐mindedness, developing between people where they trust each other and respect each other's opinions and they were the only information is brought in, is accepted between them or it can have a polar effect in which strong opinions clash and then people are more likely to believe what they originally believed. (Lewis, aged 26–32, vaccinated)

Such a situation matched the general perception of the unvaccinated cohort, who, when asked how they would feel about communicating with vaccinated people about the COVID‐19 vaccine, were open to the concept, with the caveat that the conversation must be respectful and noncombative. They described that the situation of the platform in which they had the discussion did not heavily affect their likelihood of promoting the vaccine.

I am yeah, but I mean if some people get really defensive and like especially if you say anything negative about me, I'll just back off when it when they're like I'm not trying to force people to do anything, you know? (Catherine, aged 65–72, unvaccinated)

However, a few did express a preference not to discuss vaccination, owing to the delicate nature of the conversation and the potential harm it may do to relationships. This was emphasised at various intervals of many interviews. Some of these individuals justified and explained that they maintained this perspective due to negative experiences of attempting to communicate with individuals about vaccines in the past.

Actually I keep my opinions quite close to my chest because I do recognize it is a very sensitive topic and it's a very polarising topic as well. (Jennifer, aged 26–32, unvaccinated)

### 3.3 Researching the subject

Vaccinated participants spoke about a wide array of platforms and services they used to gain information about the vaccine before becoming vaccinated. These included social media, government websites, mainstream media, primary healthcare workers and word of mouth amongst their personal and professional networks. While both vaccinated and unvaccinated individuals reported using word of mouth to find information, openness to the perspectives of people with differing opinions varied.

I was interested to hear other people's experiences with different vaccines. (Bruce, aged 18–25, vaccinated)

I probably relied on people to regurgitate some information for me. (Sophie, aged 33–40, vaccinated)

A lot of people that I associate with have this same or very similar views as me. (Sarah, aged 26–32, unvaccinated)

Across the interviews, participants frequently reported relying on one's network for new information or reinforcing existing knowledge. Many acknowledged this at various sections of the interview, suggesting that every conversation they have about vaccines ‘affects them a little bit’ and is ‘all very, very important’.

When prompted to discuss the perception of whether or not the mentioned forms of communication contributed to their decision to get vaccinated, several participants confidently responded that they were already aware that it had, which had allowed them to form an opinion on deciding to become vaccinated.

Actually I was influenced by a friend of mine that I made this year, who's a medical professional who was on the front lines and she was the one who said OK, you need to. You need to make a decision. You need to go ahead and take the vaccine. And I'm very grateful to her. Without her I probably would still be on the fence. (Nicole, aged 26–32, vaccinated)

The mention of a medical professional influencing decision‐making occurred frequently, with several participants, without prompting, mentioning a colleague, friend, or family member who are medical experts or otherwise, their family GP who was part of their close circle who contributed to their decision.

Although word of mouth presented as the most frequent form of research for individuals, participants also proactively sought out information beyond their social circles. Many described small but decisive bouts of research which included the need for specific questions to be answered. The internet was identified as a key tool for finding resources to inform decision‐making. For example:

Internet, I'll just go to Internet. I Google it first, I search, then I'll look for different [news] articles. (Linda, aged 49–56, vaccinated)

On forums and groups and actually listen to real‐life stories. (Monica, aged 33–20, unvaccinated)

Though vaccinated participants referenced a wide variety of sources that informed their research, mainstream media outlets and primary healthcare workers were frequently highlighted in varying orders of priority as a primary starting point for seeking out information. For many participants, these sources were considered more than sufficient for them to feel comfortable receiving the vaccine. Some would *‘*read some of the papers on it just to see just and inform [themselves]’ while others would go past [their] GP just beforehand just to make sure it was all good and safe and stuff’.

From the unvaccinated sample, similar findings relating to the proactive seeking out of information were established, with an emphasis on internet access. However, this sample expressed sentiments of mainstream media and scientific platforms lacking transparency and integrity. All interviewees from this group clearly articulated that they had sought out their information through independent research and conversation with individuals who shared their own opinions. There was a general sentiment of mistrust of professional and/or government agencies, which led to a stronger reliance upon individual research and word of mouth.

I've done a lot of research. I'll get a lot of backhand information, not just what's on media. I don't trust anything that media is selling. (Moira, aged 41–48, unvaccinated)

Reader of news. Avid listener of radio. Don't watch TV much, but particularly like to consume news and I consume information like there is no tomorrow. (Tom, aged 65–72, unvaccinated)

Many unvaccinated participants expressed pride and frustration in their own responses, especially in regard to the fact that they described being left with no alternative but to ‘turn towards information that was not reported by mainstream media due to conflicting information everywhere’.

Both vaccinated and unvaccinated respondents also stated that initially, they did not want or care to research the subject at all. These sentiments were driven by a variety of motivations, including a lack of care or concern for the virus, the idea that they trust what they have heard previously about vaccines or in some cases, that they are being mandated to have it administered anyway, therefore research would not make a difference. A small number spoke passionately and described a lack of patience and short‐tempered response to the topic. Some simply want to move on from speaking about the topic.

I just don't feel knowledgeable, I don't feel knowledgeable on like what is misinformation and what is correct. (Celine, aged 25–32, unvaccinated)

I'm trying to not let it be anything more than I've done the right thing. […] I've followed the health orders and I've gotten vaccinated to protect myself and my family from the virus. (Stacey, aged 33–40, vaccinated)

Vaccinated members of this cohort of participants also exhibited discomfort about having needed to become vaccinated. Many included in their initial introduction that they had become vaccinated due to work or for another logistically relevant reason, as opposed to personal choice or a desire to be better protected against the vaccine.

## 4 DISCUSSION

By conducting semi‐structured interviews with community members who either had received the COVID‐19 vaccine or had elected to remain unvaccinated, we were able to gain a rich understanding of the motivations of vaccinated individuals to communicate about the COVID vaccines, as well as how peer‐to‐peer communication potentially played a role in the decision‐making processes. Our study results echo the findings of a previous study which suggest the effectiveness of peer‐to‐peer communication in promoting good health practices.^2^, ^5^

A key finding of our study suggests there is an increased desire by individuals to promote vaccines and correct any misinformation as a direct result of becoming immunised themselves. One such explanation for this change in desire may be a difference in engagement with individual versus collective framing. By definition, individualism defines the concept of the core unit being the individual, which is supported by the society they live in, whereas collectivism is defined as the core unit being the group, and individuals fitting into the society.^19^ In harnessing an individual's desire to support their local community and its members, the use of champions or ambassadors have been used to support other health promotion endeavours. For example, the Cancer Council in South Australia engages in the use of Ambassadors who are ‘regular South Australians to want to make a difference in their community’ by promoting various healthy practices aimed at reducing cancer rates in the community.^20^ In addition, the state has also instituted health champions devoted to reducing sun safety and substance abuse.^5^

Conversely, other participants described themselves as remaining reluctant to promote the vaccine due to a desire to simply complete their civil duty and move on. Participants spoke about their preferences concerning who they would be willing to talk to and how they would share their stories about vaccination. The findings suggest that individuals would confidently relay a factual recount of their story of becoming immunised to anyone. This fear of causing a social disturbance was described as leading individuals to resist speaking to or promoting the COVID‐19 vaccine, despite an increase in motivation to do so.

Furthermore, the findings highlighted that an individual's decision‐making processes relied on a unique combination of external and internal network factors and information sources. As per the results, it was found that individuals rely upon a mixed methods approach when considering vaccination, including outward factors (media, promotional messages, etc.) and inward factors (seeking out private conversations with peers, private research, etc.). There was a consensus across most vaccinated participants that their decision and action to vaccinate with the COVID‐19 vaccine resulted from a perceived understanding that doing so protects themselves and benefits their community in different ways. This perception was not shared in the unvaccinated cohort, who viewed this same situation from a highly disparate viewpoint. This is not a new finding, with such polarising standpoints being observed in other studies.^21^ The findings also suggested that people drew from a wide array of sources, depending on their perceived trustworthiness, to make a decision on whether to vaccinate or not.

There are risks with peer communication, especially in online spaces where mis‐ and disinformation are easily disseminated. This may be due to the increasing prevalence of mis‐ and disinformation easily accessible online or through social media apps and networks.^22^, ^23^ As fact‐checkers at a community, national and international level move in haste to respond, dissect and discredit emerging sources of incorrect and harmful rumours regarding the vaccine, the effects of misinformation may also be contributing to the occurrence of vaccinated people not wanting to overstep and unvaccinated people experiencing sentiments of dismissal.^23^

While there are resources for healthcare professionals to support and improve their communication with vaccine‐hesitant patients, support materials for community members is somewhat lacking.^24^ This lack of resource provision is coupled with, as previously explored, a deficit in understanding regarding how the learnings made about improving communication efforts in a top‐down setting can be applied in a peer‐to‐peer setting. During the COVID‐19 pandemic, there was some action to engage with community‐based initiatives which empower individual championships, including the UNICEF Vaccine Ambassadorship programme.^22^ However, there is still a need for more general resources for people to communicate on a peer‐to‐peer level.

Our findings suggest that some individuals who are vaccinated have the potential to support existing vaccine promotional campaigns as peer‐to‐peer advocates, though not all feel empowered to do so. For this reason, further investigation and potential development of tailored and accessible resources to support and further empower these individuals regarding how to engage with vaccine‐hesitant people and what communication styles to use is recommended. Further research is needed to determine if this may serve to improve health outcomes and immunisation uptake in hard‐to‐reach communities. Studies have indicated that there is success in the use of champions across the health sector, with the capacity of such individuals to facilitate the adoption of evidence‐based interventions.^25^ Within the immunisation space, this may include opportunities for newly vaccinated individuals to receive information about championing the promotion of the COVID‐19 vaccine.

Other scenarios where peer‐to‐peer communication may prove as an effective communication intervention are during or immediately after a negative incident relating to a vaccine rollout. One example may be the confusing period of the Astra Zenica Vaccine rollout, where conflicting information was emerging relating to its safety. Another may be when there an individual within a community experiences a negative side effect and special attention to reiterating the rare nature of such an occurrence would be beneficial.

### 4.1 Limitations

The following are noted as limitations for this work: (1) interviews were only undertaken with a select group of participants, so the possibility of other important themes emerging cannot be ruled out; (2) due to the highly subjective, controversial and stigmatised nature of the topic, there is the potential for individuals to have felt the need to perform for the interviewer or place emphasis on points that did not align with their true feelings or opinions. This cohort was a self‐selecting group with internet access, meaning this cohort maintained motivation to partake in the study and excluded people without internet access. Lastly, the authors acknowledged that they may have inadvertently and without conscious knowledge, brought their own biases into the analysis phase, although this was consciously avoided to the best of their abilities.

### 4.2 Conclusion

The vaccinated Australian adult population may constitute an important part of the continued effort to achieve high vaccination rates amongst its population due to their increased desire to promote the COVID‐19 vaccine and correct any misinformation about it. Data from this qualitative study also provisionally suggest that those who are vaccinated feel that they are doing the right things, for their community and for themselves, while the direct opposite is considered by those who are unvaccinated. All individuals reported making use of many different platforms when seeking to have their questions relating to the vaccine answered. Further research to investigate how public health officials may be able to harness the overwhelming majority of Australians more effectively to improve health outcomes for communities around Australia and potentially, the world is encouraged.

## CONFLICT OF INTEREST STATEMENT

The authors declare no conflicts of interest.

## ETHICS STATEMENT

Approval for this study was provided by the UNSW Ethics Committee (HC: 210615). Participants were informed that their contribution was voluntary, and they could refuse to answer any question or terminate the interview at any time. All participants provided authorisation of the use of data for research purposes only. During data collection, researchers emphasised that any questions about immunisation should be discussed with a healthcare professional.

## Supporting information

Supplementary information.

## Data Availability

Data may be made available on request.
