# Supplementary material for: ‘Getting the vaccine makes me a champion of it’: Exploring perceptions towards peer‐to‐peer communication about the COVID‐19 vaccines amongst Australian adults
Source: Health Expect. 2023 May 3;26(4):1505–13. doi: 10.1111/hex.13751 (PMC10349257; doi:10.1111/hex.13751)
Supplement: Supplementary file 1 — Supplementary information. [file HEX-26--s001.docx]

***Interview Guide (HC210615)***

| ***Vaccinated Participants*** | ***Unvaccinated Participants*** |
| --- | --- |
| *Tell me a little about yourself*  *Tell me a little about your past experiences with getting vaccinated*  Do you feel that your personal experiences relating to the pandemic has changed your perceptions towards vaccines?  *How do you feel about the COVID-19 vaccine?* Probe: Have you received the COVID-19 vaccine, if so when? | |
| *Take me through how you decided to get the COVID-19 vaccine*  Probe:   1. Did you have any questions or concerns before receiving it? | Take me through about your current thinking about the COVID-19 vaccine  Probe:  1. Who has recommended that you should have it?  2. Did you have any discussions with anyone?  3. What has made you decide to not receive it? |
| Before you got vaccinated, did you look for any information about the vaccine?  Did you speak to any family members or friends?  Probe: Can you give examples about the information you looked at or what you spoke about with family/friends? | Has any of your family members or friends received the COVID-19 vaccine?  Probe:  1. What do you think about that?  2. Does it have any impact on what you think?  Have you looked for any information about the vaccine? If so, where? |
| *What are your expectations about the COVID-19 vaccine?*  Probe:  1. What effect do you believe the COVID-19 vaccine is having on society?  2. How do you think getting the COVID vaccine has changed things for you personally? | Have you been speaking to any family members, friends, or colleagues about your decision? |
| After getting vaccinated, have you spoke about your experiences with anyone?  Probe  1. Who did you speak to?  2. What did you talk about?  3. What was their reaction? Do you think you affected their perceptions towards getting vaccinated? | If you were happy to have a conversation about the COVID-19 vaccine, who would you talk to?  Probe:  1. How do you feel about talking to health workers  2. How do you feel about talking to your family or friends?  3. Is there anyone else you would be happy to chat with?  *If no, who do you talk to about health issues in general. If parent, ask about how being a parent* |
| Do you feel there has been a change with your desires or motivations tom (more confident)?  1. talk about the COVID-19 vaccine  2. correct any misinformation about the COVID-19 vaccine  If no, why?  If yes, who are you happy to talk to? Are there people you would not talk about the vaccine with?  If speaking to an unvaccinated individual about the topic of becoming immunised from COVID-19, have you thought about what you would say? Has the contents of what you’d say change since getting vaccinated? | If they were encouraging you to get vaccinated, what would you say?  Have you or would you be willing to listen to an individual who has been vaccinated about the topic of vaccination?  What would need to occur before considering vaccination?  Do you think the method and tone of communication affects the likelihood that you will become vaccinated? |
| What role do you think talking to friends and family about vaccination has on vaccination rates on you and more broadly?  Probe:  How does it compare to communication messages from health workers, government, community leaders. etc | |
| During the pandemic, what has been your experience with discussing with others about public health interventions? Mask wearing, social distancing, lockdown etc | |
| Do have anything else you would like to add that the questions may not have told me?  Thanking them for time | |
